# Solidification of Lead Ions Through Supersulfated Cement: Hydration and Mechanisms

**DOI:** 10.3390/ma19071327

**Published:** 2026-03-27

**Authors:** Fang Deng, Xiaoyan Geng, Guanjun Han, Xiaoyu Wan, Ziyu Zhou, Wendie Duan, Ling Tao, Dan Zheng, Qunpeng Cheng, Yishun Liao

**Affiliations:** 1 School of Environmental and Biological Engineering, Wuhan Technology and Business University, Wuhan 430065, China; dengfang@wtbu.edu.cn (F.D.); 15244021686@163.com (X.G.); m17371279727@163.com (Z.Z.); 15342939619@163.com (W.D.); taoling@wtbu.edu.cn (L.T.); zhengdan@wtbu.edu.cn (D.Z.); 2 School of Ecological and Environmental Sciences, Chengdu University of Technology, Chengdu 610059, China; 3 School of Urban Construction, Wuhan University of Science and Technology, Wuhan 430065, China; 15537510364@163.com (G.H.); 18014609231@163.com (X.W.); 4 School of Chemical and Environmental Engineering, Wuhan Polytechnic University, Wuhan 430073, China; cqp627@126.com; 5 Hubei Provincial Engineering Research Center of Urban Regeneration, Wuhan 430065, China

**Keywords:** phosphogypsum, supersulfated cement, lead ion, leaching toxicity, environmental protection

## Abstract

**Highlights:**

- The effect of Pb^2+^ on hydration and properties of supersulfated cement (SSC) were studied.
- Pb^2+^ can delay hydration exothermic peaks and reduce the early compressive strength of SSC.
- Pb^2+^ leaching rises with dosage yet remains within limits, confirming effective solidification.
- Solidification is achieved through adsorption, precipitation, and encapsulation in the SSC matrix.

**Abstract:**

As an extremely toxic heavy metal, lead is difficult to be degraded in the environment, and its curing and disposal is a key challenge in environmental pollution control. In this study, supersulfated cement (SSC) prepared from phosphogypsum, granulated blast furnace slag powder, and slaked lime as raw materials was used as curing cementitious material, and the curing effect and curing mechanism of SSC on lead ions were investigated by adopting testing methods such as compressive strength, electrical resistivity, X-ray diffraction (XRD), scanning electron microscopy (SEM), heavy metal ion leaching toxicity analysis, and ion concentration analysis of pore solutions. The results show that with an increase in Pb^2+^ concentration, the compressive strength of the SSC-cured paste gradually decreased, the electrical resistivity was obviously reduced, and the generation of hydration products was inhibited. The microanalysis results show that the microstructure of the cured paste became loose, and the concentration of lead ions in the SSC leach solution gradually increased, but it was much lower than the limit value stipulated in Chinese standards.

## 1. Introduction

The green transformation of the cement industry has focused on reducing cement consumption and improving the utilization of industrial waste driven by the urban renewal and environmental protection policies in China [1]. However, the industrialization process has simultaneously generated substantial hazardous wastes, including lead-containing residues. Lead (Pb) is a highly toxic heavy metal that poses severe risks to ecosystems and human health. These pollutants primarily originate from industrial solid waste and wastewater discharge, the stacking of ores and tailings in ore washing plants [2], discarded storage batteries, as well as diffuse sources like automobile exhaust and coal combustion.

It tends to migrate and spread through various pathways in the natural environment, exacerbating environmental pollution and resource waste. Against this backdrop, supersulfated cement (SSC), as a low-carbon and environmentally friendly cementitious material, has become a research hotspot in the resource utilization of industrial solid waste and the treatment of heavy metal pollution. In addition, due to the large quantity and wide coverage of industrial solid waste, it not only damages the ecological environment but also seriously affects human health and is one of the main obstacles to the in-depth implementation of the sustainable development strategy [3].

Phosphogypsum is an industrial by-product gypsum discharged from the wet-process phosphoric acid production, with an annual output exceeding 50 million tons [4] and a cumulative stockpile of over 800 million tons. Currently, phosphogypsum is mainly disposed of through stockpiling or landfilling, which causes great pollution to the surrounding ecological environment and wastes land resources [5,6]. Since the content of CaSO_4_·2H_2_O in phosphogypsum exceeds 85% [7], it is feasible to use it in the preparation of SSC, which helps to promote the resource utilization of phosphogypsum [8] and achieve the goal of resource utilization of industrial solid waste.

Supersulfated cement is a low-carbon hydraulic binder primarily composed of ground granulated blast-furnace slag (GGBFS), activated by sulfate and a small amount of alkaline components. Compared with ordinary Portland cement (OPC), SSC exhibits lower hydration heat and superior resistance to aggressive environments, particularly sulfate attack, making it a promising sustainable alternative binder [9,10]. The hydration of SSC is dominated by the formation of ettringite (AFt) and C–S–H gel, while calcium hydroxide (CH) is present only in limited amounts, contributing to improved chemical stability [9,10]. However, SSC generally shows slower early hydration kinetics, characterized by a prolonged induction period and delayed strength development, which limits its wider engineering application [10].

Cementitious materials are widely used for the immobilization of heavy metals due to their ability to reduce contaminant mobility through physical encapsulation and chemical stabilization mechanisms [11,12,13]. Among various heavy metals, Pb^2+^ is of particular concern because of its high toxicity and strong interaction with hydration processes. Previous studies have demonstrated that Pb-containing compounds can significantly retard cement hydration, prolong the setting time, and modify hydrate formation, thereby affecting both mechanical properties and durability. In addition, recent research has shown that phosphogypsum–slag-based cementitious systems can effectively immobilize Pb while maintaining acceptable engineering performance, indicating their potential for sustainable solidification/stabilization (S/S) applications.

Despite these advances, the existing literature mainly focuses on the general immobilization performance of cement-based materials, whereas the systematic influence of Pb^2+^ dosage on the hydration behavior and microstructural evolution of SSC systems remains insufficiently understood. In particular, the coupled effects of Pb^2+^ on hydration heat release, strength development, electrical resistivity, and phase assemblage in phosphogypsum-based SSC systems have not been comprehensively clarified.

Therefore, the practical objective of this study is to evaluate the feasibility of using SSC derived from industrial solid wastes for the immobilization of Pb under different Pb(NO_3_)_2_ dosages. The mechanistic objective is to elucidate the influence of Pb^2+^ on hydration behavior and microstructure evolution through a combination of macroscopic performance tests and microstructural characterization techniques, including X-ray diffraction (XRD) and scanning electron microscopy (SEM). This study aims to establish a clearer relationship between Pb dosage, hydration response, and immobilization performance in SSC systems.

## 2. Experiments

### 2.1. Materials

Ground granulated blast-furnace slag (GGBFS) with a density of 2.88 g/cm^3^ and specific surface area of 433 m^2^/kg was obtained from Xibaipo New Energy Co. (Shijiazhuang, China). Phosphogypsum (PG) has a pH value of 5.8. The phosphogypsum was first dried at 40 °C and then screened with 80-mesh molecular sieves for further utilization. The chemical composition of GGBFS and PG is shown in Table 1. Calcium hydroxide (Ca(OH)_2_) was used as an alkaline activator. Lead nitrate (Pb(NO_3_)_2_) was used as the source of Pb ions.

The dosage of Pb(NO_3_)_2_ (0–0.20 wt.%) was selected to simulate a range of Pb contamination levels commonly encountered in industrial solid wastes and contaminated soils treated by cement-based stabilization/solidification (S/S) technologies. Previous studies have reported that Pb concentrations in contaminated matrices can vary widely depending on the pollution source, and low-percentage additions in cementitious systems are typically used to represent realistic environmental exposure levels while ensuring measurable effects on hydration and immobilization behavior. It should be noted that excessive Pb^2+^ may retard cement hydration and prolong the setting time; therefore, the upper limit was restricted to 0.20 wt.% to avoid severe hydration inhibition while maintaining environmental relevance. The mix proportions of the samples are shown in Table 2.

### 2.2. Test Methods

**Figure 1:**
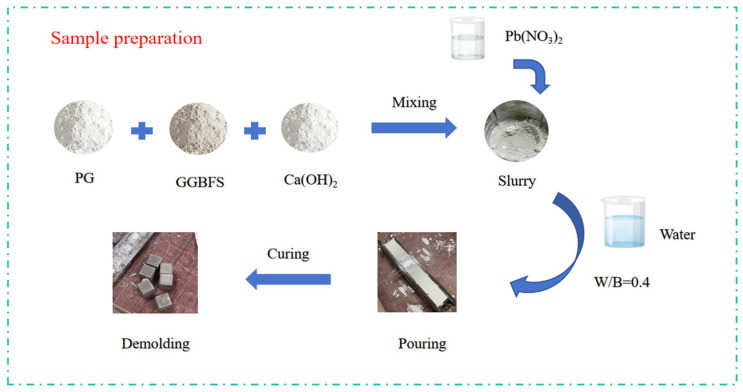
Schematic of the sample production.

The preparation process includes the following steps, as shown in Figure 1: (i) PG was dried at 40 °C and sieved to ensure a uniform particle size; (ii) GGBFS, PG, and calcium hydroxide were weighed and dry-mixed, where calcium hydroxide was added as slaked lime (Ca(OH)_2_ powder) and directly incorporated into the dry mixture; (iii) lead nitrate (Pb(NO_3_)_2_) was added in dissolved form by preparing an aqueous solution, which was used as part of the mixing water; (iv) the solution and remaining water were added at a fixed water-to-binder ratio (W/B = 0.4), followed by mechanical mixing to obtain a homogeneous slurry; (v) the paste was cast into 40 mm × 40 mm × 40 mm molds; and (vi) specimens were demolded after initial setting and cured under controlled conditions until testing.

**Figure 2:**
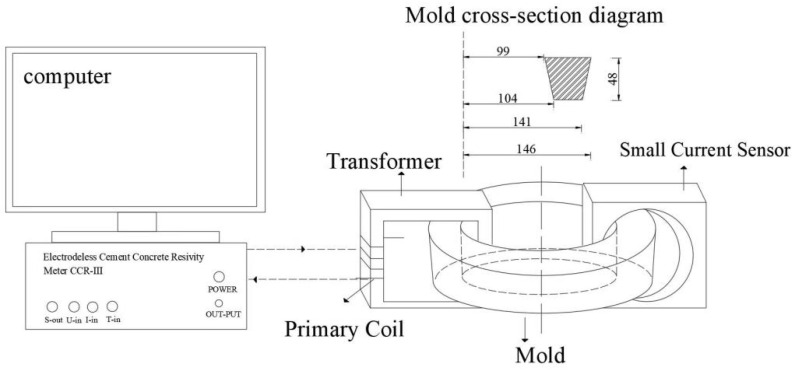
Schematic of the non-contact electrical resistivity apparatus [15].

The hydration heat was measured by an eight-channel microcalorimeter (Thermometric TAM Air), according to the Chinese standard GB/T 12959-2024 [14]. An electrical resistivity test was carried out using the electrode-free resistivity testing system of model CCR-3, as shown in Figure 2 [15]. The data was recorded every minute and lasted for 72 h. To assess the chemical environment within the cement matrix, the pore solution pH of the cement samples was determined by the conventional grinding and dissolution method. At each curing age, the internal block of the sample was ground into a powder. The powder was then screened through a 0.15 mm sieve. Then 10 g of the powder was added to 100 mL of distilled water and stirred for 30 min. After standing for 2 h, the pH value of the supernatant liquid was measured by a pH meter.

The leaching toxicity of Pb was evaluated using a standard solid-phase leaching procedure. Cement pastes specimens with dimensions of 40 mm × 40 mm × 40 mm were prepared and cured for 3 days before demolding. After demolding, the specimens were sealed and returned to the curing chamber until the target curing ages of 3 d, 7 d, and 28 d were reached. At each curing age, the hardened cement blocks were crushed and passed through a 9.5 mm Teflon sieve. Approximately 150 g of the crushed material was then placed in a 2 L extraction bottle.

The leaching solution was prepared by adding a 2:1 mixture of concentrated sulfuric acid and concentrated nitric acid to 1.5 L of distilled water, and the pH was adjusted to 3.20 ± 0.05 to obtain the extraction solution [16,17]. The leaching solution was then added to the extraction bottle containing the crushed sample.

The bottle was tightly sealed and fixed on a reciprocating shaker. The extraction was conducted at 23 ± 2 °C with a shaking speed of 30 ± 2 r/min for 18 ± 2 h. After shaking, the suspension was filtered through a 45 μm membrane filter, and the filtrate (leachate) was collected and stored at 4 °C prior to analysis [14]. The concentration of Pb in the leachate was determined using graphite furnace atomic absorption spectrophotometry (GFAAS). The analyzed ionic species was Pb^2+^. This procedure follows the Chinese standard HJ/T 299-2007 [18] and represents a leaching test performed on crushed solid samples, rather than a direct measurement of pore solutions in hardened cement paste.

The compressive strength of the cement (3 d, 7 d and 28 d of age) was tested in accordance with the method of the Chinese standard GB/T 17671-2021 by a YAW-300C-type testing machine [19]. Cement samples of 40 mm × 40 mm × 40 mm were prepared and cured at a constant temperature and in a humidity box for 3 days before demolding.

The phase composition of the sample for 3 days and 28 days was analyzed by X-ray diffraction (XRD) using a D8 Advance XRD instrument (Bruker, Karlsruhe, Germany). The scanning range was 5° to 50°, and the scanning speed was 5 °/min. After the test, the phase composition was identified using the HighScore software, version 3.0.5. The micro-morphological characteristics of the SSC at two curing ages, 3 days and 28 days, were observed using a JSM-IT300 scanning electron microscope (Hitachi, Tokyo, Japan) equipped with an X-MaxN20 energy-dispersive spectrometer.

## 3. Results and Discussion

### 3.1. Hydration Heat

**Figure 3:**
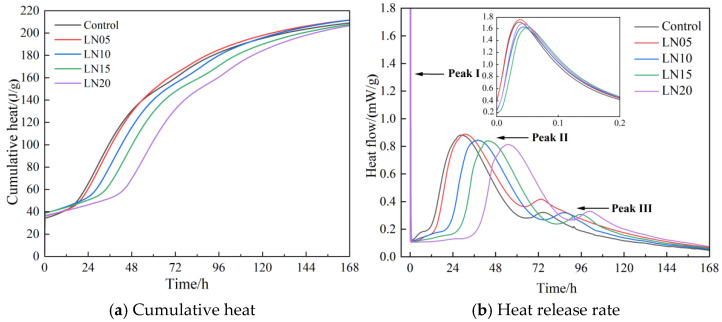
Influence of Pb^2+^ on the hydration heat of SSC cement paste.

The effects of different contents of Pb ions on the heat of hydration of the SSC at different ages are shown in Figure 3. The exothermic capacity of the cement pastes first increased and then decreased with an increase in Pb^2+^ concentration (Figure 3a). The highest exothermic capacity of the cement paste (211.64 J/g) appeared with the addition of 0.05% Pb^2+^. The variation in exothermic capacity was related to the effect of lead ions on the hydration reaction. When the addition of lead ions reached 0.1%, it had an inhibitory effect on the hydration reaction, which reduced the production of hydration products, resulting in a decrease in released heat. The initial exothermic capacity of the cement paste originated from the dissolution of raw materials, which caused the first exothermic peak (Figure 3b). Meanwhile, after the addition of Pb^2+^, the hydration exothermic rate in the induction period showed a significant decrease. Pb^2+^ could be adsorbed on the surface of slag particles to form a physical barrier, which hindered the dissolution process of slag, resulting in a reduction in heat generation in the initial stage. The effect of Pb^2+^ on the subsequent exothermic peak was reflected in the exothermic rate and appearance time. The second exothermic rate decreased from 0.879 mW/g to 0.813 mW/g, with the increase in Pb^2+^. The appearance time of the peak was delayed from 29 h to 55 h, which indicates that the Pb^2+^ could significantly slow down the hydration reaction of the cement. This could also be proven by the third exothermic peak, which was delayed from 75 h to 102 h. The hydration heat peak time of samples are shown in Table 3.

### 3.2. Electrical Resistivity

**Figure 4:**
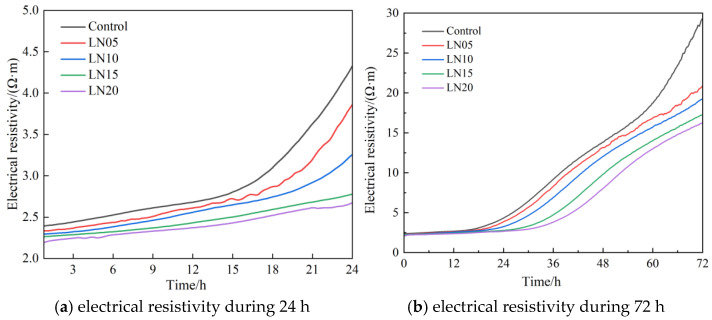
Influence of Pb^2+^ on the electrical resistivity of SSC cement paste.

The effect of Pb^2+^ on the electrical resistivity of the SSC at different ages is shown in Figure 4. The results show that the electrical resistivity of the cement paste decreased with an increase in Pb^2+^ dosage, while the induction period of cement hydration was prolonged. The electrical resistivity at 3 d decreased from 29.51 Ω·m to 16.28 Ω·m with the Pb^2+^ dosage increasing from 0% to 0.2%. The OH^−^ in the system could make a great contribution to the electrical conductivity of the cement paste pore solution [20]. Pb^2+^ was easy to react with the OH^−^ in the system to produce lead compound precipitation, which reduced the alkalinity of the system and made the electrical conductivity decrease. Hence, the resistivity of each sample increased gradually during the 3 days.

Moreover, the electrical resistivity of cement paste is negatively correlated with porosity [20]. The products generated during the hydration process could fill the initial pores, which made the system gradually dense, while Pb^2+^ could be preferentially adsorbed on the surface of the cement particles or the growth sites of hydration products, which hindered the reaction between the water and reactive ions. The increase in porosity caused a delay of the hydration reaction and a prolongation of the hydration induction time.

The evolution of electrical resistivity reflects changes in ionic transport properties and pore structure development in the cementitious system during hydration. In cement-based materials, electrical resistivity is mainly controlled by the connectivity of the pore network and the concentration of ions in the pore solution. At early hydration stages, the pore structure of fresh cement paste is relatively open and contains a high concentration of dissolved ionic species, resulting in relatively high electrical conductivity and therefore low electrical resistivity [21].

As hydration proceeds, the formation of hydration products, such as ettringite (AFt) and calcium silicate hydrate (C-S-H), gradually refines the pore structure and reduces pore connectivity. Meanwhile, the hydration reactions consume dissolved ions from the pore solution, which lowers the ionic concentration within the pore network. The combined effects of pore refinement and ion consumption reduce ionic transport in the pore system, leading to a gradual increase in electrical resistivity with curing age [22].

The incorporation of Pb^2+^ may influence this evolution by affecting the hydration kinetics of the SSC system. Previous studies have reported that certain heavy metal ions can interact with reactive surfaces of cement particles or early hydration products, which may inhibit slag dissolution and delay hydration reactions to some extent. Consequently, the formation of hydration products may be slowed, resulting in a less refined pore structure at early curing ages.

In addition, changes in pore solution chemistry may also influence electrical conductivity. Variations in alkalinity and ionic concentration affect the mobility of charge carriers within the pore solution. When hydration reactions are delayed, relatively higher concentrations of dissolved ions may remain in the pore solution, which contributes to higher electrical conductivity and therefore lower electrical resistivity at early ages [21].

Overall, the evolution of electrical resistivity in the SSC system reflects the combined effects of hydration progress, pore structure development, and pore solution ionic concentration. Therefore, electrical resistivity measurements can provide indirect insights into the microstructural development and hydration behavior of the cementitious matrix.

### 3.3. Mechanical Properties

**Figure 5:**
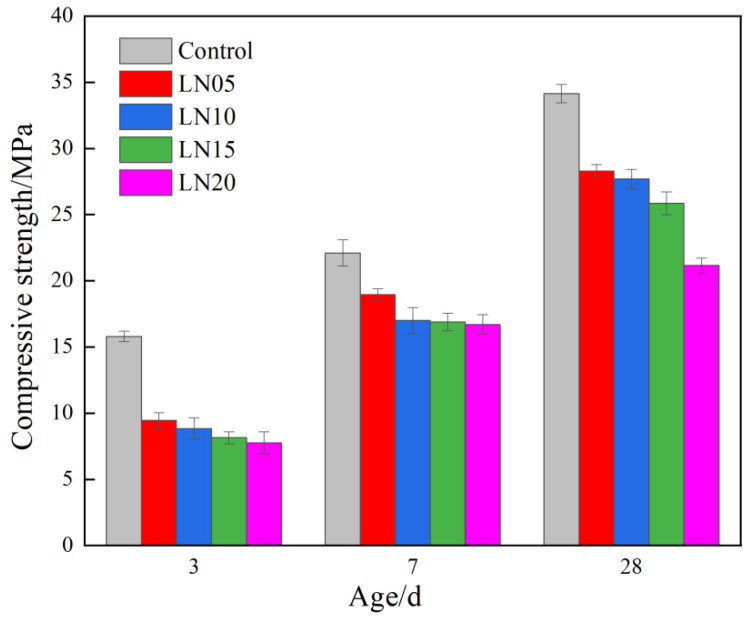
Influence of Pb^2+^ on the compressive strength of SSC cement paste.

Figure 5 illustrates the influence of Pb^2+^ on the compressive strength of the SSC at the different curing ages. The compressive strength of all specimens increased with a prolonged curing time. Compared to the strength at 3 d, the compressive strength of the various samples at 7 d increased by 33.8%, 111%, 92.4%, 108.4%, and 102.4%, respectively. Subsequently, the 28 d strengths showed further improvements of 57.9%, 46.4%, 58.2%, 50.9%, and 31.9% relative to the corresponding 7 d values. These results indicate that the early-age strength development in Pb-containing SSC follows a more pronounced growth trend.

A comparison between the LN20 and LN05 samples reveals that the incorporation of a higher Pb^2+^ content led to reductions in compressive strength of 9.9%, 13.5%, and 22.1% at 3 d, 7 d, and 28 d, respectively. This suggests that the adverse effect of increased lead ion concentration becomes progressively more significant with an extended curing age. Specifically, the compressive strengths of LN05 were 9.45 MPa, 18.95 MPa, and 28.3 MPa at 3 d, 7 d, and 28 d, respectively, while those of LN20 decreased to 8.15 MPa, 16.9 MPa, and 25.85 MPa at the corresponding ages.

In comparison with the control sample without the addition of lead nitrate (compressive strengths of 15.8 MPa, 22.1 MPa, and 34.15 MPa at 3 d, 7 d, and 28 d, respectively), the LN05 specimens exhibited strength reductions of 43%, 10.3%, and 16.9% at the same curing ages. These findings demonstrate that the incorporation of lead nitrate exerts a noticeable detrimental effect on the compressive strength of SSC, with the most pronounced impact occurring at early ages.

The underlying mechanism can be attributed to the following: Under the combined activation of phosphogypsum and the alkaline environment, reactive SiO_2_ and Al_2_O_3_ components in GGBFS react with gypsum to form ettringite and C-S-H gel. However, the presence of Pb^2+^ ions inhibits the hydration reactions of the cementitious system, thereby reducing the formation of hydration products [16,23]. Since early-age strength development relies heavily on the rapid generation of these hydration products, the inhibitory effect of Pb^2+^ is most significant during the early stages. As hydration progresses over time, this inhibition is partially mitigated, resulting in a reduced magnitude of strength loss at later ages.

### 3.4. Leaching Toxicity of Lead Ion

**Figure 6:**
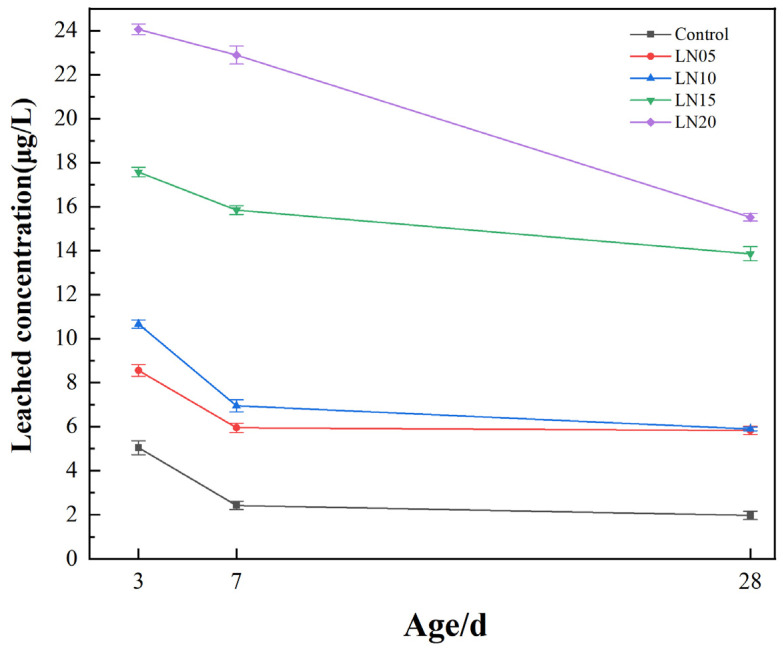
The lead ion leaching toxicity of SSC cement paste.

The Pb^2+^ leaching toxicity at different ages of the SSC is shown in Figure 6. It can be seen that the leaching toxicity of Pb^2+^ gradually decreased with an increasing age, while the leaching toxicity gradually increased with an increase in Pb^2+^ dosage. The leaching solution concentrations of Pb^2+^ in the LN05 sample at the different ages were 8.56 μg/L, 5.95 μg/L, and 5.83 μg/L, respectively, at 3 d, 7 d, and 28 d, which increased by 69.5%, 145.9%, and 185.8% compared with the control sample. This indicates that the addition of Pb^2+^ had a significant impact on the lead ion leaching toxicity of SSC cement. These observations suggest that immobilization may involve multiple pathways, including adsorption, precipitation, and physical encapsulation, although direct evidence is beyond the scope of the present study.

Meanwhile, the Pb^2+^ leaching concentration of SSC cement at 3 d was 24.06 μg/L, while it decreased to 15.52 μg/L at 28 days. It was still far below the lead concentration limit in leachate specified in the “Identification Standards for Hazardous Waste —Identification for Leaching Toxicity”(GB 5085.3-2007) [24], which is specifies a maximum allowable concentration of less than 5 mg/L for Pb^2+^. The leaching results show that the Pb^2+^ concentration in the leachate increases with an increasing Pb^2+^ dosage but decreases progressively with curing age. This trend indicates that the immobilization capacity of the SSC matrix improves as hydration proceeds. Similar age-dependent reductions in heavy-metal leaching have been reported in phosphogypsum–slag cementitious systems, where the formation of hydration products and matrix densification play key roles in reducing metal mobility [1,16].

SSC mainly consists of GGBFS, sulfate activators, and alkaline components. During hydration, thedissolution of GGBFS releases reactive Ca, Si, and Al ions, which subsequently react with sulfate ions to form AFt and C–S–H gel, the primary hydration products responsible for the development of strength and microstructure in SSC systems [25].

As hydration proceeds, AFt crystals and C–S–H gel progressively fills capillary pores and interweave to form a denser solid skeleton, resulting in pore-size refinement and reduced pore connectivity. The resulting compact microstructure restricts the transport pathways of dissolved Pb^2+^ ions and therefore decreases their leaching potential [26].

In addition to physical encapsulation by the hardened matrix, chemical immobilization may also contribute to Pb stabilization. Pb ions can be adsorbed or structurally incorporated into C–S–H gel, while precipitation processes may occur under alkaline conditions when the Pb^2+^ concentration is relatively high [27].

Furthermore, phosphogypsum often contains residual phosphate species that may react with dissolved Pb to form sparingly soluble lead-phosphate minerals, which can further reduce Pb mobility in cementitious systems [28].

This indicates that SSC cement had good solidification capacity for Pb^2+^. Based on the literature, the immobilization of Pb^2+^ in cementitious systems primarily involves four mechanisms: physical adsorption by the C-S-H gel [29]; chemical fixation via ion exchange with SO_4_^2−^ and Cl^−^ in calcium aluminate [30,31]; chemical precipitation as insoluble heavy metal salts with anions present in the pore solution [32]; and physical encapsulation within the dense matrix of the hardened SSC paste [33]. 

### 3.5. Microstructure

#### 3.5.1. pH Value

**Figure 7:**
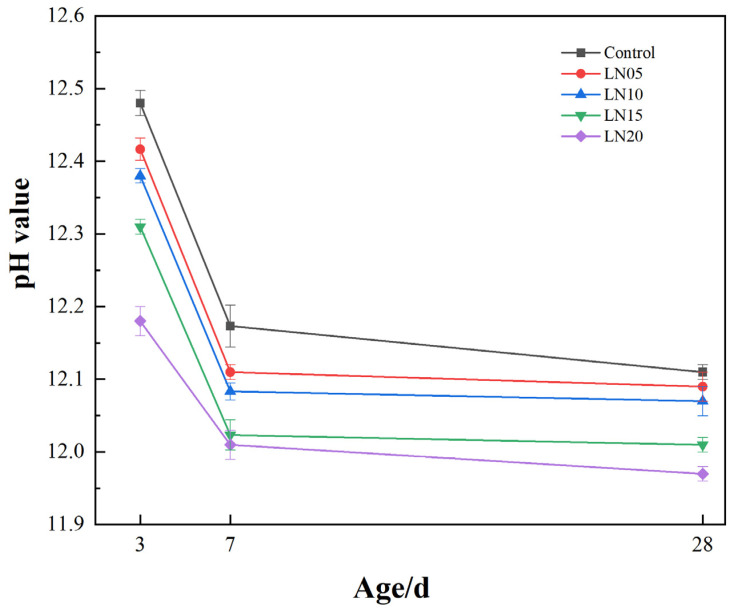
The pH value of the pore solutions for SSC cement.

The variation in pore solution pH in SSC systems reflects the evolution of hydration reactions and ionic equilibria within the cement matrix. The effect of Pb^2+^ on the pore solution pH of the SSC at the different ages is shown in Figure 7. It can be seen that the pH of the SSC in each sample decreased with a growth in age. This behavior indicates that the hydration reactions proceeded rapidly in the early stage and gradually stabilized at later ages.

At the early stage of hydration, the dissolution of alkaline activators, such as Ca(OH)_2_, releases a large amount of OH^−^ into the pore solution, producing a strongly alkaline environment. Such alkaline conditions are essential for activating slag particles and promoting the dissolution of reactive Si and Al species from the glassy slag structure [34]. Previous studies have shown that sufficient alkalinity significantly enhances slag hydration kinetics and facilitates the formation of hydration products in SSC cement systems [35].

As hydration proceeds, the dissolved Ca^2+^ and Al species react with sulfate ions to form ettringite (AFt), while silicate species participate in the formation of C-S-H gel. The continuous precipitation and accumulation of these hydration products consume Ca(OH)_2_ and modify the chemical equilibrium of the pore solution, leading to a gradual decrease in pH with curing age [36]. Meanwhile, the progressive densification of the hydration structure further restricts ion transport and stabilizes the pore solution chemistry.

The incorporation of Pb^2+^ resulted in a slight reduction in pore solution pH compared with the control sample. This phenomenon can be attributed to the interaction between Pb^2+^ and hydroxyl ions in the alkaline pore solution, which may lead to the formation of Pb-containing hydroxide or complex precipitates, thereby consuming part of the OH^−^ in the system [17,27,37]. In addition, Pb^2+^ can adsorb onto the surface of slag particles or early hydration products, which may hinder slag dissolution and delay hydration reactions to some extent, further influencing the evolution of pore solution chemistry [37,38].

#### 3.5.2. Lead Ion Concentration of Pore Solution

The effects of Pb^2+^ on the pore solution ionic concentration of the SSC are shown in Table 4. The Pb^2+^ concentration in the pore solution decreased with an extension in age. The Pb^2+^ concentration at 3 d decreased by 81.4%, 53.4%, 41.5%, 44.0%, and 23.9%, respectively, compared with that at 28 d. This phenomenon was mainly attributed to two aspects: (1) with the extension in age, the C-S-H gel and calcium aluminate continued to fill the pore system, causing a decrease in porosity [39] and a reduction in the fluidity of the pore solution. The physical restriction enhanced the role of the lead ions wrapped in the closed pore space, inhibiting the migration and diffusion of the lead ions; (2) the hydration of products such as C-S-H gels generated by hydration increased, resulting in an increase in their surface adsorption sites, which could continuously capture Pb^2+^ through physical adsorption, thus leading to a decrease in Pb^2+^ concentration. The concentration of lead ion of pore solution in SSC cement paste is shown in Table 4.

C–S–H gel possesses a large specific surface area and abundant adsorption sites, which provide favorable conditions for interactions with heavy metal ions. Recent studies have shown that Pb^2+^ can interact with C–S–H through surface adsorption, ion exchange, or structural association, depending on the Ca/Si ratio and the structural characteristics of the gel [27,38,40].

Meanwhile, the densification of the hydration matrix reduces pore connectivity and restricts ion diffusion within the pore system, which further limits the migration of Pb species [27,41]. However, when the Pb^2+^ dosage exceeds the immobilization capacity provided by hydration products, part of Pb^2+^ may remain in the pore structure in a relatively mobile form.

In addition, the concentration of Pb^2+^ in the pore solution showed an increasing trend with an increase in the Pb^2+^ dosage. When the dosage reached 0.2%, the Pb^2+^ concentration significantly increased compared with the control sample, which was due to the fact that the SSC system could be Pb^2+^ partially cured by hydration products, such as C-S-H gels. Meanwhile, there was a limit to the capacity of the curing. Even if the aging period was prolonged, the curing space for Pb^2+^ by the new hydration products was limited. The excess Pb^2+^ could not be completely fixed, resulting in an increase in free lead ion concentration in the whole solution. However, the concentration of Pb^2+^ was still within the limit value of lead concentration in leachate in the Chinese standard GB 5085.3-2007 (Pb^2+^ < 5 mg/L).

#### 3.5.3. Hydration Products

**Figure 8:**
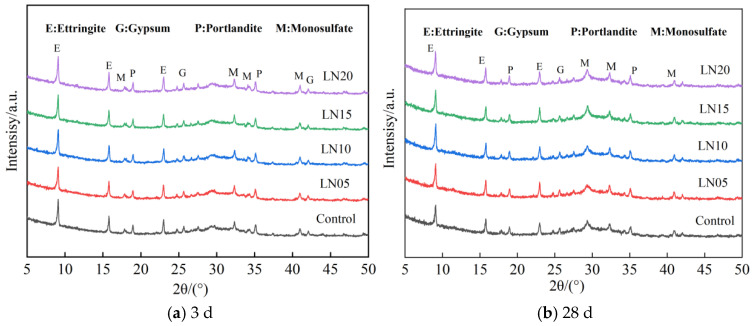
XRD patterns of the hydration products of SSC cement paste at different curing ages.

XRD patterns of the cement pastes cured at 3 d and 28 d are shown in Figure 8. Several diffraction peaks were observed, corresponding to calcium aluminate phases, gypsum, calcium hydroxide, and a possible AFm-like phase. The main crystalline phases in the SSC system are AFt and gypsum, while C–S–H mainly exists as an amorphous phase. Similar phase assemblages have been widely reported in SSC cement and phosphogypsum–slag-based cementitious systems [42,43,44]. These hydration products are formed through the activation of slag in the presence of sulfate and alkaline species, leading to the precipitation of AFt and the formation of C–S–H gel during hydration [35,45].

With an increasing Pb^2+^ dosage, the diffraction intensity of some hydration products slightly decreases, indicating that Pb^2+^ may influence the hydration process of SSC. Previous studies have suggested that heavy metal ions can interact with reactive surfaces of cement particles and hydration products, which may inhibit slag dissolution and delay the nucleation and growth of hydration products [37,46,47]. This effect can reduce the formation rate of AFt and C–S–H at early ages, thereby influencing the development of the cementitious matrix.

Furthermore, the formation of AFt and C–S–H plays a key role in the immobilization of heavy metals in cement-based systems. These hydration products can provide adsorption sites and structural frameworks that contribute to the stabilization of metal ions [13,48]. Although XRD results mainly reveal the evolution of crystalline phases, they cannot directly determine the chemical state or structural location of Pb within the hydration products.

**Figure 9:**
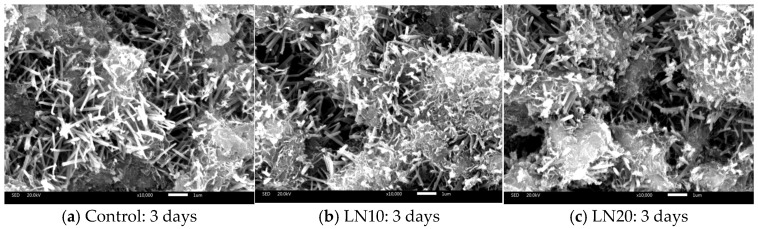

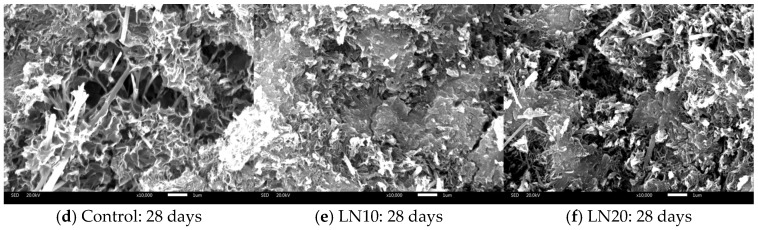
SEM images of different samples cured for 3 and 28 days.

SEM images of the cement cured after 3 d and 28 d were shown in Figure 9. The hydration products of the SSC system were mainly needle-and-rod AFt and reticulated C-S-H gels [11]. SEM observations show that the microstructure of the SSC matrix mainly consists of needle-like AFt crystals and reticulated C–S–H gel. These hydration products interweave to form a three-dimensional network structure, which contributes to the mechanical strength and structural stability of the hardened matrix. Similar microstructural characteristics have been reported in phosphogypsum–slag-based cementitious systems and SSC cement materials [44,49,50].

As hydration proceeds, the continuous formation of AFt and C–S–H gradually fills the pore structure, leading to a denser microstructure. Refinement of the pore structure and a reduction in pore connectivity play a crucial role in restricting the migration of dissolved metal ions within the matrix [51]. This microstructural densification is widely recognized as an important factor contributing to the immobilization of heavy metals in cement-based materials.

However, when Pb^2+^ is introduced into the system, the microstructure becomes relatively looser, and the amount of AFt appears to decrease at early curing ages. This observation suggests that Pb^2+^ may retard the hydration process and influence the formation of hydration products. Similar microstructural changes have been reported in cement systems containing heavy metals, where metal ions can interfere with hydration reactions and modify the morphology of hydration products [27,37,52].

Therefore, the SEM observations indicate that the immobilization of Pb^2+^ is closely associated with the development of the hydration microstructure. The formation of a dense AFt andC–S–H network may contribute to the physical encapsulation of Pb^2+^ ions and limit their migration within the matrix.

## 4. Conclusions

This study investigated the solidification mechanism of lead ions in SSC prepared from industrial solid wastes, based on an analysis of macroscopic performance indicators and microscopic characteristics. The relevant conclusions are as follows:

The incorporation of Pb^2+^ exerts a dose-dependent adverse effect on the mechanical performance of SSC. Specifically, a 0.20% Pb(NO_3_)_2_ dosage resulted in a 34.9% reduction in the 28-day compressive strength compared to the control sample.

The incorporation of Pb^2+^ significantly inhibits the early hydration process of SSC. A higher Pb^2+^ dosage reduces electrical resistivity and causes the cumulative heat release to first increase and then decrease, indicating that Pb^2+^ delays the hydration reaction of SSC.

The incorporation of Pb^2+^ alters the microstructure of SSC. The content of hydration products decreases, and the microstructure becomes relatively loose at early ages. With an extension in curing age, hydration continues, and the matrix becomes denser, which is beneficial to reducing the migration of Pb^2+^.

The SSC matrix exhibits good immobilization capacity for Pb^2+^. Although the Pb^2+^ concentration in the leachate increases with an increasing Pb^2+^ dosage, it decreases with curing age and remains far below the regulatory limit. The reduction in Pb leaching is closely associated with the formation of hydration products and the densification of the microstructure. The immobilization of Pb^2+^ in the SSC system is therefore likely related to combined effects, such as physical encapsulation, interactions with hydration products, and possible precipitation under alkaline conditions.

## Figures and Tables

**Table 1:** Chemical composition of raw materials (wt.%).

Material	CaO	SiO_2_	Al_2_O_3_	MgO	SO_3_	TiO_2_	Fe_2_O_3_	Na_2_O	K_2_O	P_2_O_5_	F^−^	LOI
PG	43.03	9.51	0.99	0.66	36.35	0.08	0.40	0.17	0.29	1.91	0.86	5.75
GGBFS	44.02	29.25	12.38	7.79	2.07	1.34	0.92	0.56	0.47	0.04	0.16	0.46

**Table 2:** Mix proportions of samples (wt.%).

Sample	GGBFS/%	PG/%	Ca(OH)_2_/%	Pb(NO_3_)_2_/%	W/B
Control	85	10	5	0	0.4
LN05	85	10	5	0.05	0.4
LN10	85	10	5	0.10	0.4
LN15	85	10	5	0.15	0.4
LN20	85	10	5	0.20	0.4

**Table 3:** Hydration heat peak time of samples (h).

Sample	Time for the 2nd Peak	Time for the 3rd Peak
Control	28.3	74.4
LN05	30.5	74.7
LN10	38.2	87.8
LN15	43.2	95.8
LN20	54.6	101.1

**Table 4:** The lead ion concentration of pore solution in SSC cement paste (mg/L).

Sample	3 d	7 d	28 d
Control	7.68	4.64	1.43
LN05	9.28	5.62	4.32
LN10	10.76	8.98	6.29
LN15	13.74	9.79	7.7
LN20	19.53	16.03	14.86

## Data Availability

The original contributions presented in this study are included in the article. Further inquiries can be directed to the corresponding author.
